# Intravoxel Incoherent Motion Diffusion Weighted MR Imaging for Monitoring the Instantly Therapeutic Efficacy of Radiofrequency Ablation in Rabbit VX2 Tumors without Evident Links between Conventional Perfusion Weighted Images

**DOI:** 10.1371/journal.pone.0127964

**Published:** 2015-05-28

**Authors:** Ziyi Guo, Qiang Zhang, Xiaoguang Li, Zhengyu Jing

**Affiliations:** 1 Department of Radiology, Peking Union Medical College Hospital, Chinese Academy of Medical Sciences and Peking Union Medical College, Beijing, 100730, China; 2 Department of Radiology, Haikou People’s Hospital, Xiangya Medical School, Central South University, Haikou, 570208, Hainan, P.R. China; Sainte-Anne Hospital Center, FRANCE

## Abstract

**Objective:**

To investigate the intravoxel incoherent motion diffusion weighted imaging (IVIM-DWI) as a potential valuable marker to monitor the therapy responses of VX2 to radiofrequency ablation (RF Ablation).

**Methods:**

The institutional animal care and use committee approved this study. In 10 VX2 tumor–bearing rabbits, IVIM-DWI examinations were performed with a 3.0T imaging unit by using 16 b values from 0 to 800 sec/mm^2^. The true diffusion coefficient (D), pseudodiffusion coefficient (D^*^) and perfusion fraction (f) of tumors were compared between before and instantly after RF Ablation treatment. The differences of D, D^*^ and f and conventional perfusion parameters (from perfusion CT and dynamic enhanced magnetic resonance imaging, DCE-MRI) in the coagulation necrosis area, residual unablated area, untreated area, and normal control had been calculated by compared t- test. The correlation between f or D^*^ with perfusion weighted CT including blood flow, BF (milliliter per 100 mL/min), blood volume, BV (milliliter per 100 mL/min), and capillary permeability–surface area, PMB (as a fraction) or from DCE-MRI: transfer constant (K_trans_), extra-vascular extra-cellular volume fraction (V_e_) and reflux constant (K_ep_) values had been analyzed by region-of-interest (ROI) methods to calculate Pearson’s correlation coefficients.

**Results:**

In the ablated necrosis areas, f and D^*^ significantly decreased and D significantly increased, compared with residual unblazed areas or untreated control groups and normal control groups (*P* < 0.001). The IVIM-DWI derived f parameters showed significant increases in the residual unablated tumor area. There was no significant correlations between f or D^*^ and conventional perfusion parameters.

**Conclusions:**

The IVIM-DW derived f, D and D^*^ parameters have the potential to indicate therapy response immediately after RF Ablation treatment, while no significant correlations with classical tumor perfusion metrics were derived from DCE-MRI and perfusion-CT measurements.

## Introduction

Image-guided percutaneous ablative therapies using thermal energy sources, such as radiofrequency (RF) [1], microwave [2], laser-induced thermal therapy [3], and high-intensity focused ultrasound [4], are rapidly evolving as nonsurgical minimally invasive techniques for the treatment of malignant neoplasms. Imaging of solid tumors after RF Ablation is crucial to judge the completeness of the ablation and later, to detect therapy response [5]. This assessment is usually performed with contrast-enhanced computed tomography [6] or magnetic resonance imaging [7]. When the use of contrast agents is contraindicated, assessment of the residual tumor can be a problem. In addition, assessment of the accuracy of ablation completeness by immediate CT or MR imaging has inevitable limitations, because postablation changes make an accurate diagnosis difficult [8][9]. The development of imaging tests without contrast agent is a challenge to immediately assess the treatment effects of RF Ablation.

Diffusion-weighted imaging (DWI) has been applied to measure tumor changes to assess the therapy response [10][11]. Normally, the apparent diffusion coefficient (ADC) of water molecules within living tissues is derived from diffusion images with an assumption that the water molecular diffusion is a random process [12]. This type of diffusion is also known as Gaussian diffusion and is characterized by a simple mono-exponential decay model. However, tumor tissue is a complex and restricted environment, which hinders the distribution of water molecules, resulting in distributions that are far from Gaussian diffusion [13]. DWI reflects the microscopic translational motions from the influence of both capillary perfusion and molecular diffusion that occurs within each image voxel. Increasing diffusion gradients lead to a multi-exponential signal decrease within each voxel, in agreement the intravoxel incoherent motion (IVIM) theory [14][15]. IVIM-DWI provides methods to measure the respective influence of true molecular diffusion and microcirculation within a voxel. The parameters derived from IVIM including: diffusivity (D), perfusivity (D^*^) and its volume fraction (f) values, which reflect the extent of diffusion and blood perfusion at the capillary level.

However, whether the perfusion parameters D^*^ and f correlate with standard perfusion CT and MRI measurement has not been fully illuminated. The purpose of this study was to investigate the value of IVIM-DWI, DCE-MRI, and perfusion CT techniques in assessing the response of VX2 to RF Ablation therapy, with particular emphasis on the role of IVIM-DWI as a potential valuable imaging marker of therapy response.

## Material and Methods

### VX2 tumor model

Ten New Zealand white rabbits weighing from 2.5 to 3.0 kg were used. The protocol was approved by the Committee on the Ethics of Animal Experiments of Beijing Union Medical College Hospital (Permit Number: SYXK20100028), and the study was carried out in strict accordance with the recommendations in the Guide for the Care and Use of Laboratory Animals of the National Research Council. All surgery was performed under anesthesia using sodium pentobarbital administered into the marginal ear vein. The VX2 tumor strain (National Infrastructure of Cell Line Resources, Beijing, China) was transplanted into the hind thigh muscles of tumor-carrier rabbits. The tumor-carrier rabbit was then sacrificed by an overdose of anesthesia, and the VX2 tumor was excised and placed in saline. Necrotic tissue was discarded. The tumor tissue was cut into 1-mm^3^ fragments. Two to three tumor fragments were symmetrically implanted into each side of the erector spinae of 10 rabbits under CT-guidance. After 10–14 d, both plain and contrast enhancement CT scans were performed on the 10 rabbits to evaluate the growth of VX2 tumor. The implanted tumor mass had grown to an average size of 2.0 cm.

### CT guided RF Ablation for the treatment of VX2 tumors

**Fig 1:**
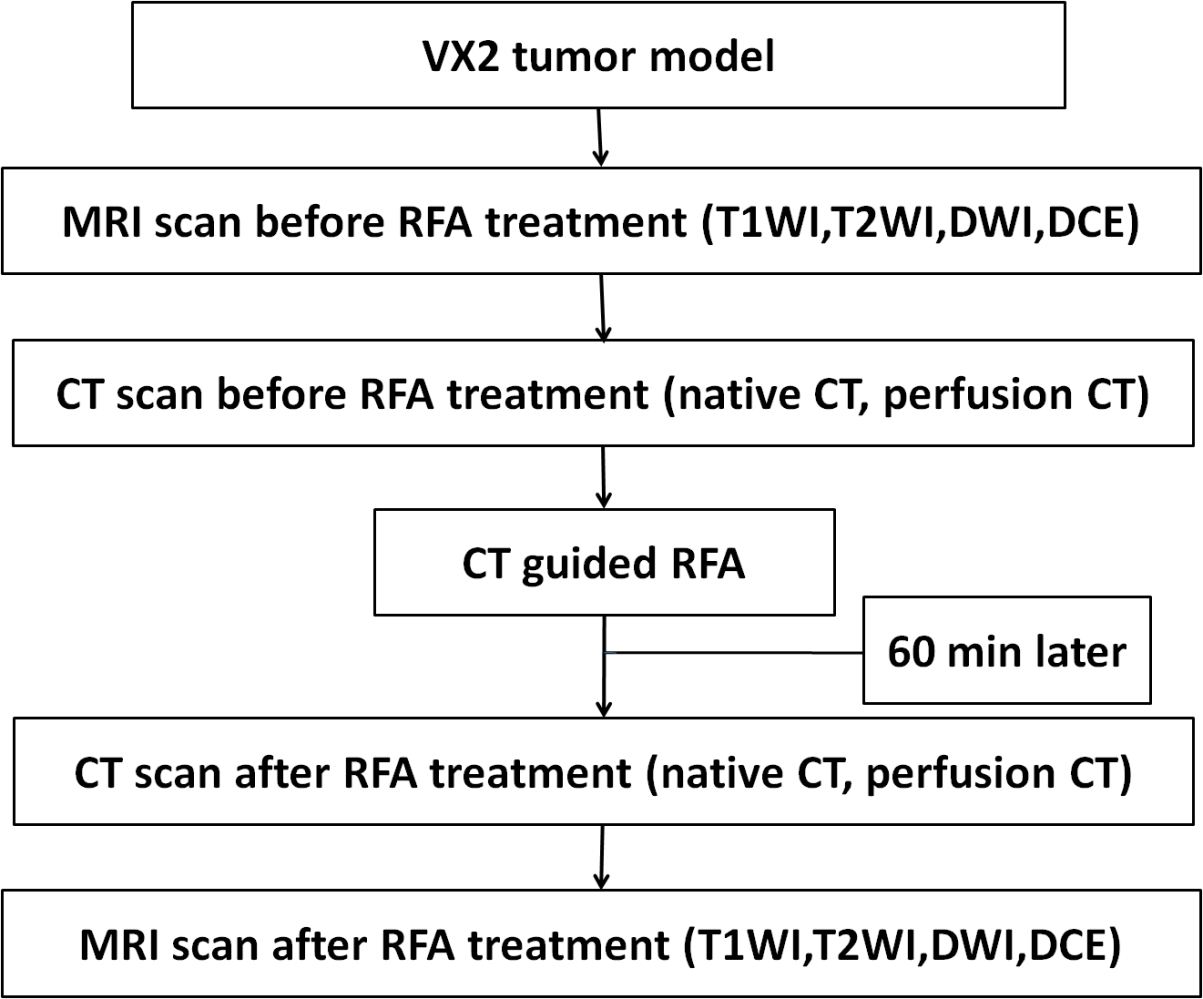
Flowchart shows the experimental procedure in used in this study.

To prepare for RF Ablation, the hair was shaved and the surgical site was disinfected using povidone-iodine. A scout scan through the tumor was obtained. Axial CT scans both plain and perfusion CT imaging had been used to select the target treatment level of the tumor. VX2 tumor ablations were achieved using a single 15-gauge internally cooled bipolar electrodes (Celon ProSurge, Berlin, Germany) with an active tip of 2.0 cm length. The RF Ablation needle (CelonProSurge, Berlin, Germany) was injected at a depth two-thirds of the target tumor’s diameter under the gauidence of CT. The applied parameters as follow: power 25W, about 5 min, total 5 KJ. The electrode was internally cooled with chilled saline circulated by a peristaltic pump. The RF system is constituted by one 200-watt power generator supplying pulsed energy automatically adjusted based upon tissue impedance during a full 5-minute ablation session. Each animal was treated once in a single setting. The diameter of RF Ablation zone was about 1cm. At least 60 min apart, allowing the contrast material from the preceding acquisition to clear, perfusion CT imaging and MRI examination was performed again to evaluate the curative effects of the RF Ablation as shown in Fig 1. The rabbits were fastened to a plastic board to ensure the same postion during the whole experiment.

### Perfusion CT Examination and Image Analysis

For lesion detection, an unenhanced scan of the entire tumor was obtained before beginning cine CT scanning. The native scan parameters were as follows: 80 kVp, 120 mAs, slice thickness 5 mm, matrix = 512×512, FOV = 150 mm, 30 slices. This was followed by a cine scan (1 phase / second) during the whole proccess of intravenous administration of contrast medium, iopromide (350 mg of iodine per milliliter, 2 mL/kg) at a rate of 2mL/sec into an ear vein via an 18-gauge catheter. Contrast medium was injected into an ear vein by using a power injector (Stellant; MEDRAD, Pittsburgh, PA). All CT examinations were performed with a multisection spiral CT scanner (Somatom Perspective; Siemens Medical Solutions, Forchheim, Germany). Cine CT scanning parameters were as follows: 120 mAs/rotation, 80 kV, 512 × 512 matrix, 150 mm FOV, section thickness/spacing of 5/5 mm, and an acquisition series of 90 time frames at 1-second intervals with 10 phases of blank scans before the injection of contrast agent. Cine CT was performed again after the CT guided RF Ablation therapy.

**Fig 2:**
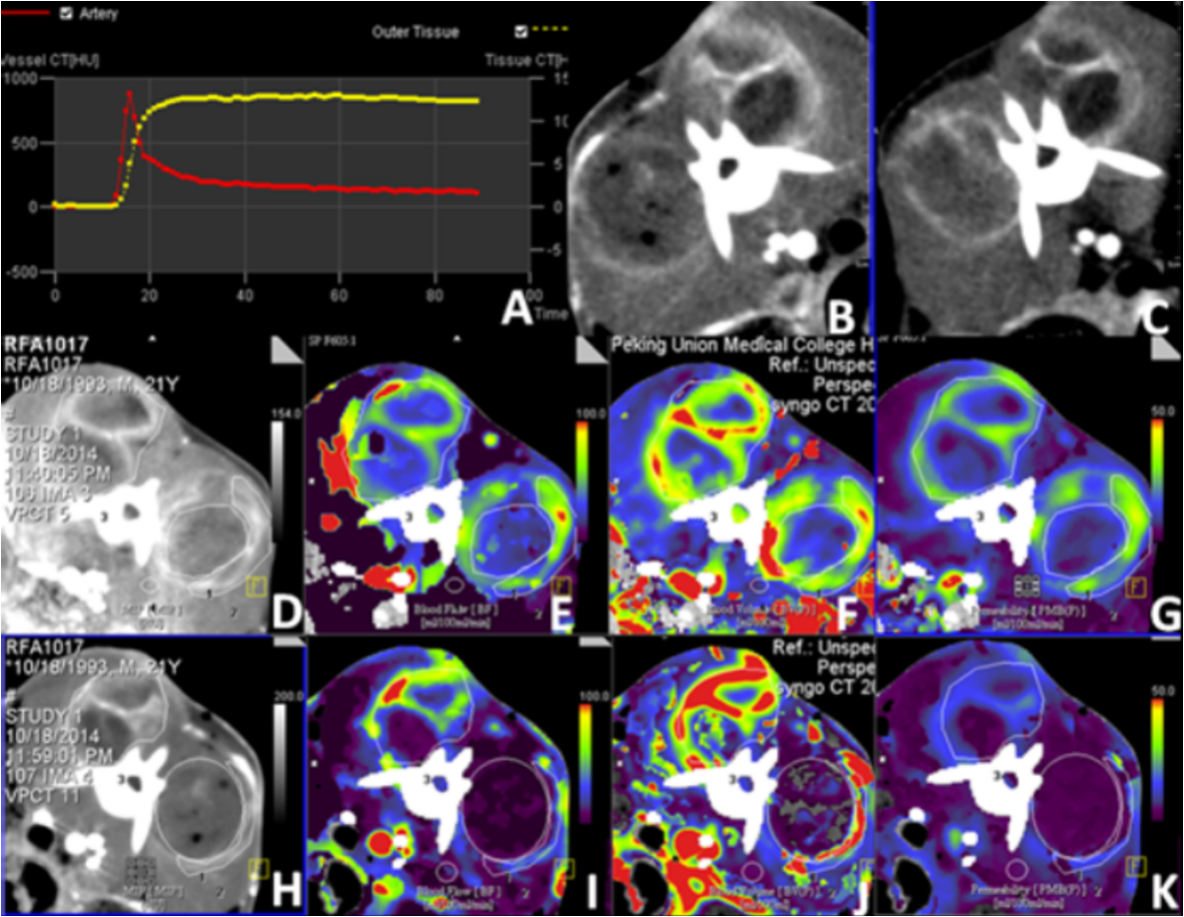
Perfusion CT derived parameter mappings of one rabbit with implanted VX2 tumor. (A) Arterial input function for the simulation curve; (B) and (C). Raw images for CT perfusion mapping after and before RF Ablation treatment. (D to G) before RF Ablation treatment (H to K) after RF Ablation treatment: (D) fused and (E) pure BF maps (F) Blood volume (unit in mL/100 mg), (G) permeability of capillary vessel surface (scale in 0.5 mL/min/100 mg) show greater enhancement values in the tumor than that of the adjacent muscle. Higher blood flow, blood volume and permeability had been observed for the tumor (arrow) compared to the adjacent muscle tissues and almost equal to contralateral tumor. (I to K) Significantly reduced values of BF, BV, and PMB in the RF Ablation treated tumor (arrow) than that of the contralateral untreated tumors.

All CT perfusion images were transferred to an image processing workstation (*Syngo via*; Siemens Healthcare, Erlangen, Germany). Tumor perfusion software (Siemens Healthcare Systems) was used for CT perfusion analysis. The software used curve-fitting by least mean squares to obtain mathematical descriptions of the time–density curves for each pixel [16]. Regions of interest were placed on tumors (RF Ablation treated necrosis areas, residual unablated areas and contralateral untreated, namely T_RF Ablation,_ T_residual_, and T_control_), and normal tissue (Normal), as shown in Fig 2B to 2E. Subsequently, functional maps had been automatically generated, including blood flow BF (milliliter per 100 mL/min), blood volume BV (milliliter per 100 mL/min), and capillary permeability–surface area PMB (as a fraction). Each pixel value had been represented with a color scale. The BF, BV, and PMB were measured in the tumors and in an area with normal muscle tissue (no tumor area) in each case by 2 senior radiologists (G.Z.Y. and Z.Q.).

The absence of early arterial hyperperfusion at the margins of the tumor was interpreted as complete remission and grouped as T_RF Ablation_. Peripheral hyperperfusion of the lesion in the early arterial hyperperfusion phase was considered to indicate residual vital unablated tumor tissue, grouped as T_residual_. After RF Ablation has been completed, rabbits were moved back to the MR scanner for post-treatment MRI.

### MRI Examination

All MR imaging examinations were performed at our institution with the same 3.0-T MR imaging system Skyra (Siemens Healthcare, Erlangen, Germany) and two 8-channel surface radiofrequency receiving coils. The MR imaging protocol included transverse T1-weighted turbo spin echo and transverse T2-weighted turbo spin echo sequences. DW images were acquired prior to gadolinium chelate injection. Finally, a time-resolved MR angiography with Stochastic Trajectories (TWIST) T1-weighted perfusion weighted sequence was repeated 75 times during the whole process of contrast injection with 5 phases of blank scans before the injection of contrast agent. The total of 0.2 mL per kilogram of body weight gadoteric acid (Omniscan TM; General Electric healthcare, USA) had been injected both before and after RF Ablation treatment. The time interval of two injections was over 1 hour to eliminate the residual effects of contrast agent. Protocol details are shown in Table 1.

**Table 1:** MRI protocols and main parameters in this experiment.

parameter	Morphological MRI		DWI	Dynamic contrast
Sequence	T2W TSE	T1W TSE	Single-shot EPI	TWIST
Orientation	Axial	Axial	Axial	Axial
TR/TE (ms)	5000/75	450/11	3000/83	4.5/1.94
Fat suppression	not	not	yes	not
FOV (mm)	150×150	150×150	150×150	150×150
Matrix	256×256	256×256	128×128	128×128
Slice thickness (mm)	5	5	5	5
Number of slices	20	20	10	20
NEX	1	1	1	1
b value (s/mm^2^)	no	no	0,50,100,150,200,250,300,350,400,450,500,550,600,650,700,800	no

Note: DWI = dffusion weighted image; TSE = turbo spin echo; TWIST = time-resolved imaging with stochastic trajectories; TR/TE = time repetition time/echo time; FOV = field of view; NEX = number of excitations.

### Image Analysis of DCE-MRI

All MRI perfusion images were transferred to an image processing workstation, *tissue 4D* software (Siemens Healthcare Systems) was used for time resolved DCE-MRI analysis. A parameter related to vessel permeability and tissue blood flow transfer constant (K_trans_), extra-vascular extra-cellular volume fraction (V_e_) and reflux constant (K_ep_) values had been calcualted. Calculation is based on the Tofts model and pharmacokinetic calculation using a 2-compartment model on a pixel-by-pixel basis [17]. Regions of interest (ROIs) were manually drawn as the same way as CT perfusion analysis. The K_trans_, K_ep,_ V_e_ had been measured in the tumors (T_RF Ablation,_ T_residual_, T_control_), and normal muscle tissue (Normal) in each case. Tumor lesions were identified by two senior radiologists (GZY and ZQ) for consensus.

### Image Analysis of DWI

The bi-exponential model was mathematically expressed as follows to analysis IVIM-DWI:

$$S\left(b\right)=S_{0}\left(f\cdot e^{-b\cdot D^{*}}+\left(1-f\right)\cdot e^{-b\cdot D}\right)$$ 1

where f is the unit of perfusion volume fraction. D^*^ is the pseudodiffusion coefficient influenced by tissue microcapillary perfusion, and D is the true diffusion coefficient. The diffusion quantification and analysis of IVIM were conducted by the MITK-Diffusion software (http://www.mitk.org/) [18].

**Fig 3:**
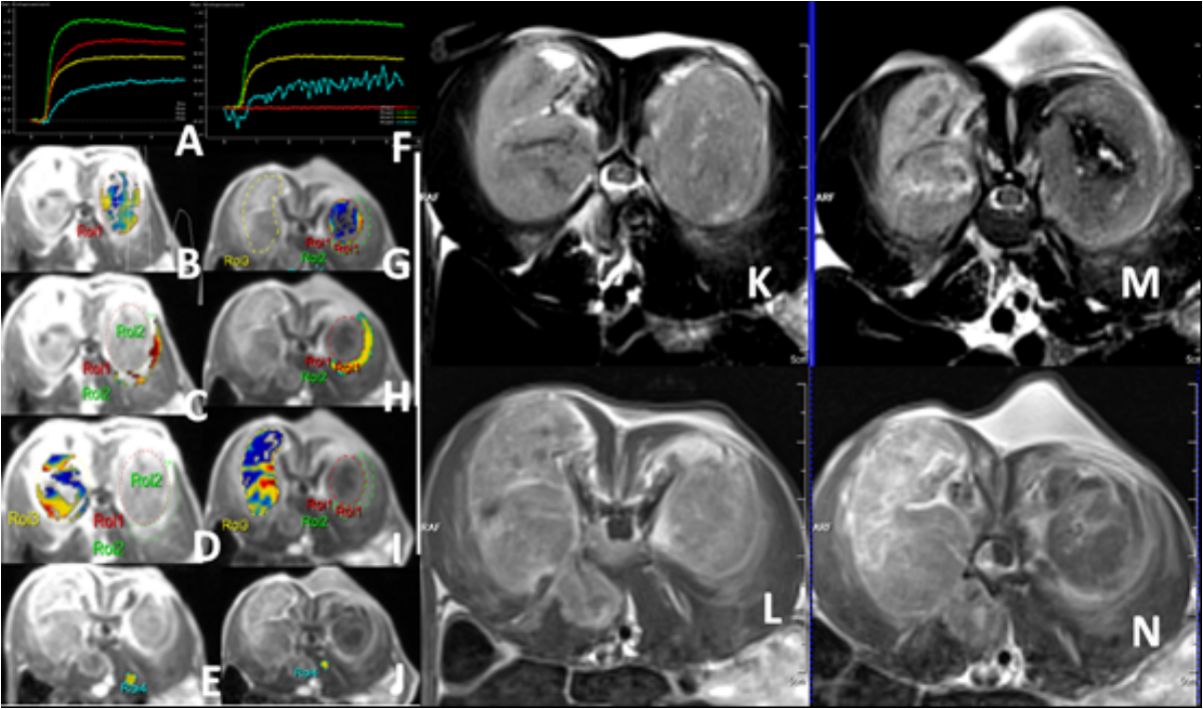
Perfusion MRI derived parameter mappings of one rabbit with implanted VX2 tumor. A. Arterial input function for the simulation curve; and (B-E) ROIs selection for T_RF Ablation_, T_residual_, T_control_ and normal regions for claculation the K_trans_, V_e_, and K_ep_ parameters before RF Ablation treatment; F-J after RF Ablation treatment corresponding to A to E. All the mapping from A to J show reduced perfusion values in the RF Ablation treated tumor than that of the contralateral untreated tumors and before treatment in color coded mappings. K to L show the T2, T1 post contrast enhancement MRI before RF Ablation treatment and after treatment (M to N). All the mapping from K to N show instantly morphological chages of routine MRI in the RF Ablation treated tumor. The T2WI and T1WI showed the necrosic focus in tumor after RF Ablation (M, N).

**Fig 4:**
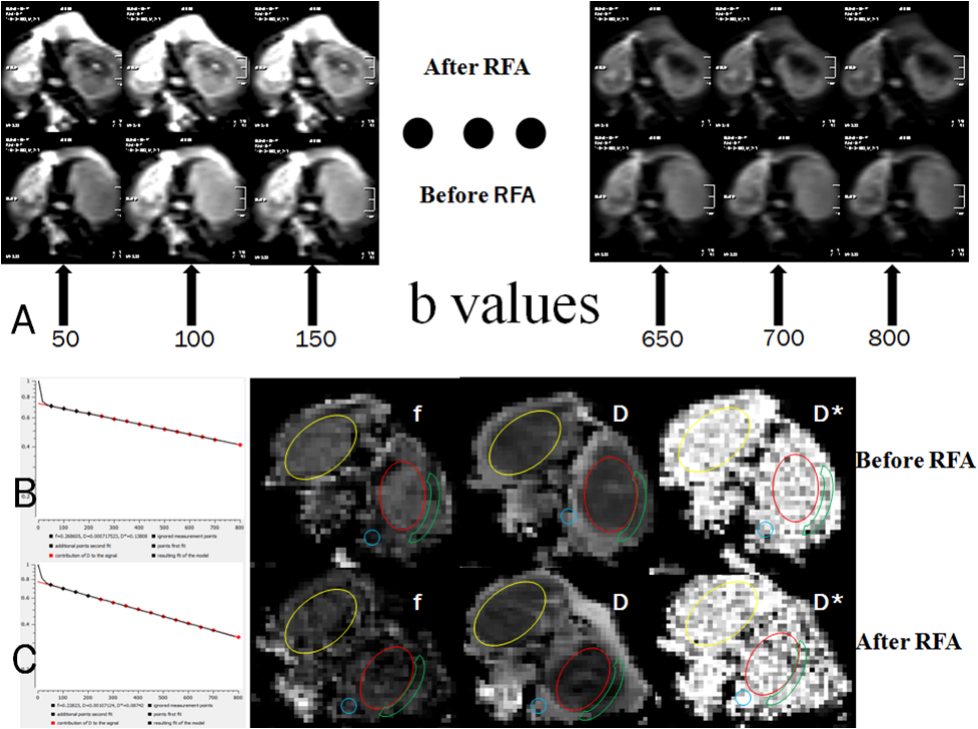
Image processing demonstrated in on rabbit bearing VX2 tumor. (A) Top row demonstrates the sequencial diffusion weighted images b value from 50 to 800, low row corresponding to the before treatment as control. (B to C) the IVIM-DWI parameters f, D and D* derived from above multiple diffusion weighted images (A). The IVIM analysis derived f, D, D^*^ map were obtained with pixel-by-pixel basis from the each pixel's signal intensity in the IVIM-DWI data. It was shown that reduced f value and increased D value indicated the RF Ablation therapy necrosis responses.

DWI datasets were analyzed in consensus by two senior radiology researchers. Each lesion observed in the DW images had also been defined on the T2W, T1W with post-contrast images, all serving as a roadmap for regions of interest (ROIs) demarcation. ROIs were drawn nearly the same methods as perfusion CT. D, D^*^, f, were calculated in each of the ROIs, as shown in Fig 3B to 3E). All the differences before and after ablation were compared to confirm any recorded changes. The typical image processing is demonstrated in Fig 4A to 4C).

### Trypan blue injection

Trypan blue (TB; Sigma-Aldrich Co., LLC, St. Louis, MO, USA) was dissolved in 10 mM phosphate buffer (pH 7.4) and sterilized by passing through 0.2 μm pore membrane filters. The final dye was 2.0% TB. Animals were injected in the ear vein with ~1.0 mL/kg body wt and then sacrificed by anesthetic overdose. The animals were visually inspected for dye uptake into the tumor, indicated by blue.

### Histopathologic analysis

The tumor and surrounding tissue were orderly harvested according to the distance from the central necrosis area. The necrosis area was confirmed by in vivo TB injection intravenously into the ear margin. All histopathologic analyses were used to gather evidence of the curative effects of thermal ablations. The specimen tissues were cut into 5.0-μm sections and prepared for histological assessment using hematoxylin and eosin staining. The histopathologic analysis was performed using microscopy by one senior pathologist.

### Statistical analyses

Comparisons between before and after treatment were analyzed using the compared t-tests for parametric continuous data of BF, BV, PMB, K_trans_, K_ep_, V_e_, D, D^*^, and f in the RF Ablation treated necrosis, residual unablated, untreatment control, and normal groups. Pearson's correlation analysis were used to evaluate the correlations between the f or D* values obtained with IVIM and BF, BV, PMB, K_trans_, K_ep_, V_e_ values obtained with perfusion CT and MRI. All these data had been analyzed by the ROI-based approach. Statistical analyses were performed using SPSS for Windows Version 16.0 (SPSS Inc., Chicago, IL, USA), and *P* < 0.05 was considered significant.

## Results

### Establishment of rabbit VX2 tumor model

Direct mass implantation tumor had 100% successful established tumors and produced two similar tumors at same level for the following tests. This direct mass implantation was suitable for evaluating RF Ablation efficacy, the implanted tumor mass grew to an average size of 2.0 cm (ranging from 1.2 to 3.0 cm) by 10 to 12 days after its introduction.

### Therapeutic effect of RF Ablation revealed by perfusion CT and DCE-MRI

Using perfusion CT and MRI technique, we evaluated a total of 10 VX2 tumor models before and immediately after treatment with RF Ablation. These lesions were located in both sides of erector spinae. RF Ablation treatment of these VX2 tumors produced an average of a 1.0 cm coagulation necrosis as shown by contrast enhancement CT and MRI. The coagulation necrosis either remained unchanged or had no contrast agent perfusion on the perfusion images, as shown in Figs 2 and 3. If the perfusion image showed a weighted portion, it meant that the tumor tissue was not completely ablated and is an indication of residual tumors. The descriptive analysis of the perfusion parameters measured for all the VX2 tumor models showed the following results (Table 2 and 3). The conventional perfusion parameters including BF, BV, PMB, K_trans_, V_e_, and K_ep_ were all significantly reduced (*P* < 0.001) in the T_RF Ablation_ group, which indicated the therapy effects of RF Ablation on tumor. And the V_e_, in the T _residual_ significantly increased (*P* < 0.001) after RF Ablation treatment indicated the alterations of vascular exchage fuction alterations instantly after RF Ablation therapy.

**Table 2:** Compare perfusion parameters in the necrosis area, residual unablated, contralateral untreated tumor, and normal muscle before and after RF Ablation.

parameters	T_RF Ablation_		T_residual_		T_control_		Nomal	
	Before	After	Before	After	Before	After	Before	After
BF	33.48±18.12	0.87±1.87^**^	33.31±9.92	35.91±3.33	34.20±16.34	33.10±8.54	0.56±0.15	0.03±0.27
BV	5.61±1.10	0.93±1.10^**^	4.91±1.17	8.03±2.72^**^	5.66±1.88	5.88±1.49	3.98±0.67	3.41±0.57
PMB	17.72±7.97	0.68±1.2^**^	15.53±3.22	16.23±6.57	17.37±7.30	16.18±6.29	1.06±0.75	0.91±0.43
K_trans_	0.13±0.05	0.02±0.02^**^	0.11±0.05	0.14±0.09	0.13±0.12	0.11±0.10	0.05±0.01	0.04±0.02
V_e_	0.29±0.08	0.06±0.71^**^	0.28±0.06	0.49±0.02^**^	0.31±0.14	0.30±0.16	0.18±0.02	0.17±0.04
K_ep_	0.50±0.14	0.12±0.22^**^	0.49±0.12	0.51±0.09	0.53±0.20	0.49±0.18	0.20±0.05	0.24±0.27

n = 10.

Note:

^1. *^ *P*<0.05

^**^ *P*<0.01

2. T_RF Ablation_ = necrosis area treatment by RF Ablation, T_residual_ = residual unablated tumor, T_control_ = control side untreatment by RF Ablation, Nomal = normal muscle tissue, BF = blood flow, BV = bllod volume, PMB = permeability surface–area product, K_trans_ = transfer constant, V_e_ = extra-vascular extra-cellular volume fraction and K_ep_ = reflux constant values.

**Table 3:** Compare IVIM parameters (D, D^*^, f) in the necrosis area, residual unablated, contralateral untreated tumor, and normal muscle, n = 10.

Parameters	T_RF Ablation_		T_residual_		T_control_		Nomal	
	Before	After	Before	After	Before	After	Before	After
f	0.29±0.05	0.15±0.04^**^	0.29±0.04	0.42±0.03^**^	0.31±0.03	0.30±0.02	0.12±0.04	0.12±0.02
D (10^–3^)	0.61±0.15	0.92±0.04^**^	0.75±0.15	0.79±0.40	0.70±0.01	0.71±0.01	0.94±0.03	0.90±0.02
D^*^	0.13±0.03	0.06±0.02^**^	0.12±0.09	0.11±0.08	0.11±0.07	0.10±0.08	0.11±0.06	0.11±0.04

Note:

1. ^*^ p<0.05

^**^ p<0.01.

2. T_RF Ablation_ = necrosis area treatment by RF Ablation, T_residual_ = residual unablated tumor, T_control_ = control side untreatment by RF Ablation; Nomal = normal muscle tissue, D = true diffusion coefficient, D^*^ = pseudodiffusion coefficient, f = perfusion fraction.

### Therapeutic effect of RF Ablation revealed by IVIM-Diffusion weighted imaging

A summary of three IVIM parameters for each group there were measured before and immediately after treatment with RF Ablation is presented in Table 3. The D values had significantly increased, f and D^*^ value measured in the tumor were significantly reduced in the T_RF Ablation_ group induced by RF Ablation therapy (*P*<0.001). The f value in the T_residual_ was significantly increased in the T_residual_ group after RF Ablation therapy (*P*<0.001) (Table 3). There were not significant changes in the other groups including T_control_ and Normal before and after RF Ablation treatment.

### IVIM-Diffusion and perfusion CT and MRI Correlation

Correlation between perfusion parameters derived by perfusion CT and DCE-MR measurements and IVIM-based parameters are summarized in Table 4. No significantly correlations of f or D^*^ to conventional perfusion parameters had been found.

**Table 4:** Correlation coefficients and cooresponding p values between perfusion parameters derived from perfusion measurements and IVIM-based perfusion parameters (D^*^, f).

Parameters	T_RF Ablation_		T_residual_		T_control_		Notrmal	
	D^*^	f	D^*^	f	D^*^	f	D^*^	f
BF	0.12 (0.65)	0.15(0.21)	0.32(0.23)	0.23(0.87)	-0.14(0.31)	0.11(0.57)	0.29(0.53)	0.27(0.43)
BV	0.27(0.43)	-0.12(0.61)	0.19(0.21)	0.26(0.54)	0.21(0.64)	0.31(0.35)	-0.11(0.26)	0.15(0.09)
PMB	0.09(0.21)	0.21(0.45)	0.29(0.27)	0.30(0.21)	0.23(0.65)	0.37(0.76)	0.23(0.34)	0.21(0.13)
K_trans_	0.14(0.24)	-0.29(0.21)	0.25(0.19)	-0.21(0.11)	0.19(0.87)	0.31(0.11)	0.22(0.29)	0.22(0.19)
V_e_	0.17(0.98)	0.09(0.09)	-0.14(0.23)	0.23(0.43)	0.11(29)	0.25(0.15)	0.21(0.79)	0.24(0.68)
K_ep_	0.19(0.76)	0.32(0.68)	0.21(0.33)	0.34(0.21)	0.27(54)	0.22(0.32)	0.29(0.21)	0.13(0.75)

Note:

^*^ p<0.05

^**^ p<0.01.

2. T_RF Ablation =_ necrosis area treatment by RF Ablation, T_residual =_ residual unablated tumor, T_control =_ control side untreatment by RF Ablation; Nomal = normal muscle tissue, D^*^ = pseudodiffusion coefficient, f = perfusion fraction, BF = blood flow, BV = bllod volume, PMB = permwability, K_trans_ = transfer constant, V_e_ = extra-vascular extra-cellular volume fraction and K_ep_ = reflux constant values.

### Histopathologic results

**Fig 5:**
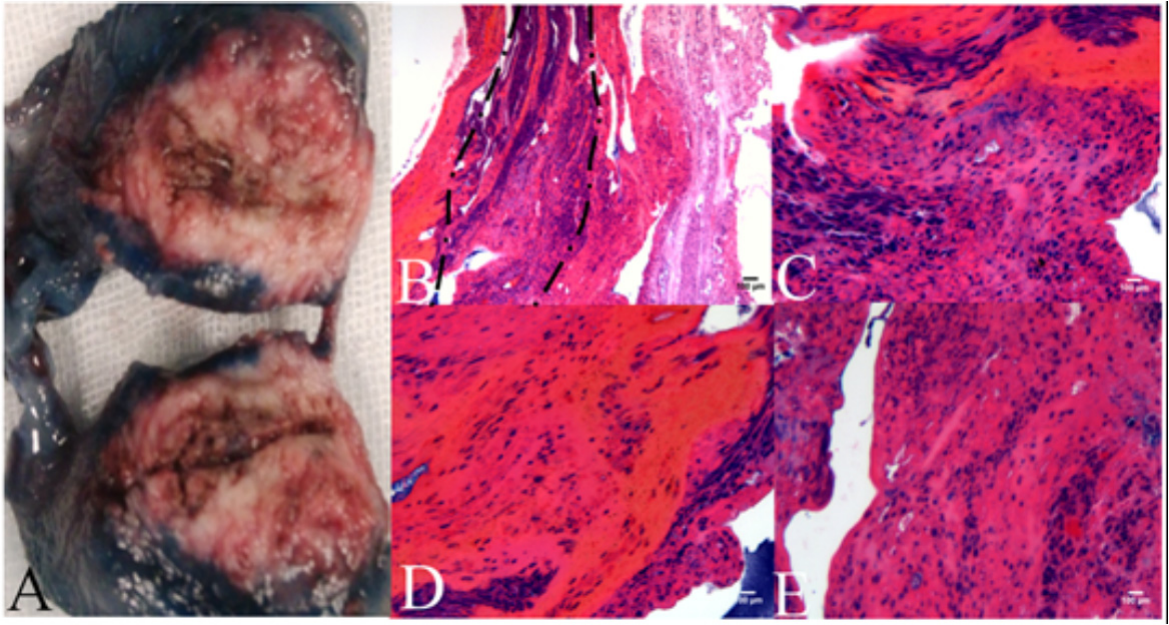
Histopathological photos of the hepatic VX2 tumor and adjacent muscle tissue. (A) Viable cells exclude Trypan blue (TB), while dead cells stain blue from TB uptake. TB staining of VX2 tumor after in vivo treatment shows damage to the tissue structure within the area occupied by the RF Ablation. VX2 tumor treated with RF Ablation *in vivo*, its curative effects had been verified by pathology Hematoxylin/eosin staining. (B) Region near the right side demonstrates areas near the RF Ablation needle and shows coagulation necrosis areas, X 100. (C) Region demonstrates nucleus of tumor cells was partially dissolved and necrotic, X 200. (D to E) Regions demonstrate transition from total coagulation necrosis to residual unablated tumor (partial necrosis), X 200.

The rabbits in the treatment group were sacrificed immediately after RF Ablation treatment for histologic studies. For hemotoxylin-eosin–stained tumor specimens, ablation zones were observed to encompass the transitions from necrosis—residual unablated- normal tumor within samples collected immediately after RF Ablation. The ablated zones within tumor were pale relative to adjacent residual unablated tissue regions, indicating necrosis, away from the necrosis zone (Fig 5A). Partial loss of tissue was found at the center of the electrode insertion for the rabbit showing complete tumor ablation, and this was surrounded by a pale coagulated necrotic area and a darker portion with venous TB stain. The VX2 cells appeared to have undergone coagulation necrosis in a large area, and nucleus condensation, karyorrhexis, and karyolysis could be seen in individual cells. Coagulation necrosis is characterized by preservation of the basic cell outlines of necrotic cells (Fig 5B to 5E).

## Discussion

The major goals of this work were to evaluate the usefulness of IVIM-diffusion weighted, imaging-based methods to evaluate the therapeutic response of RF Ablation on VX2 tumors model. In our study, direct tumor mass implantation forms a spherical tumor, which is similar to the naturally occurring tumor. For RF Ablation treatment in this experiment, we set the RF energy at total 5KJ (25 W, about 5 minutes) and to ensure high consistency and repeatability of the therapeutic range. An average 1.0 cm focus is formed using these parameters. CT guidance is needed during RF Ablation treatment to verify tumor location and avoid tissue damage. Because the electrode had been inserted from the dorsal lateral side to the target tumor, the postion remain stable and without motion artifacts make the study of perfusion CT or MRI, IVIM-DWI and their relationship simple and repeatable.

We demonstrated that IVIM-DWI is able to depict therapy responses in a rabbit VX2 tumor model after administration of RF Ablation treatments, as shown in Fig 4. Among the IVIM-DW imaging parameters, relative changes in D*, D, and f can be utilized as predictors of tumor response immediately after RF Ablation. In the RF Ablation treated group, perfusion-related IVIM parameters, including D* and f immediately decreased, whereas diffusion related parameter D increased at the same time. Our findings in this study are consistent with previous study about the early evaluation of the treatment response after RF Ablation of unresectable lung tumours by DWI [19]. These results suggest that pre- and post-RF Ablation IVIM-DW can be used to predict the therapeutic outcome before the change in tumor size becomes detectable on CT or MRI images. In addition, perfusion-related IVIM parameters immediately increased in the residual tumor tissue, which is consistent with the changes of V_e_ and BV from conventional perfusion-weighted imaging.

One retrospective cohort study of MRI data included both pre- and post- laser interstitial thermal therapy (LITT) T2WI and DWI acquisitions, which yielded an Apparent Diffusion Co-efficient (ADC) map. Their initial results indicate that the ADC maps demonstrated highly sensitive, albeit non-specific, response to LITT-related changes in vivo, due to the significant change in water permeability in tissue as a result of treatment [20]. One in vivo canine study indicated that ADC may be a better tool to predict microwave-induced tissue necrosis than contrast-enhanced imaging, including DCE-MRI, and contrast enhanced ultrasound (CEUS) [21]. There is a substantial change in perfusion after MR-guided high-intensity focused ultrasound ablation of uterine fibroids due to vessel destruction. Low b-values ADC might be the good choice to emphasis perfusion changes in ADC maps [22]. Most recently, intravoxel incoherent motion (IVIM) diffusion-weighted (DW), magnetic resonance (MR) imaging has been used to evaluate the diagnostic value of quantitative assessment of the therapeutic efficacy of a vascular disrupting agent (VDA) (CKD-516) in rabbit VX2 liver tumors [23]. In this study, the authors derived the f and D^*^, in addition to D, which reflects the perfusion-insensitive water diffusion in tissues by performing biexponential IVIM model fitting to the multiple–b value DW imaging data [24]. Our current results and the conclusions from previous studies indicated that IVIM-DW could be used to obtained tumor perfusion information on the capillary level relevant to RF Ablation treatments and may be useful to evalue the reaction of tumor after RF Ablation treatments.

Another purpose of this study was to explore whether the IVIM-based D^*^ and f metrics measured tissue perfusion by perfusion CT or DCE-MRI, and thus may deliver diffusion and perfusion information simultaneously without the need for intravenous, contrast media based on this VX2 tumor model. Many authors have indicated that IVIM-DW may be an alternative (or even superior) to DCE-MRI and could either link or correlate D^*^ and f values with relative cerebral blood volume in brain [25–27] and modeling of the tumor vasculature in prostate tracer [28]. For this reason, we had examined VX2 tumor models by means of IVIM, DCE-MRI, and perfusion CT imaging, both before and after RF Ablation treatment, in the same experiment. Our current results indicate that the correlation coefficient between perfusion parameters derived from perfusion CT or DCE-MRI and IVIM-based perfusion parameters are low (r never above 0.4), irrelevant (r less than 0.2), or negative in most instances, as shown in Table 4. Our findings are in agreement with other authors’ conclusion regarding no clearly evident link between D^*^, f, and DSC- and DCE-derived metrics [29][30].

A cause of these findings may have been the low signal-to-noise ratio of D^*^ estimates in VX2 tumor, the extensively altered hemodynamics and challenge to model capillary properties in tumor tissue, and the more complex than assumed nature of origin for the biexponential signal decay in the IVIM-DW measurements; this does not share the same principles as the intravascular tracer behavior [31]. In addition, it is worth noting that the kinetic model used in DCE-MRI or perfusion CT is only valid for tissues that contain small blood volumes [32], so the parameters might not be accurate for VX2 tumors because VX2 tumors have a larger blood volume?. It was believed that the IVIM method to measure perfusion remains technically challenging; for example, the optimal fitting algorithm and the optimal distribution of b values, as well as the accuracy and robustness of the measurements [33]. Overall, the experimental verification of the relation between the IVIM perfusion parameters and the standard perfusion parameters are still lacking.

This study had several limitations. First, we only evaluated the immediately changes of RF Ablation treatment. Thus, we could not assess late histologic changes or tumor recurrence that might have explained the late changes in IVIM parameters and its relation to conventional perfusion parameters. Second, to measure IVIM parameters, we selected one ROI on one axial MR image of the tumor. However, considering the three-dimensional structure of the tumors, measurement on cross-sectional images might be less representative of the entire tumor than volumetric measurements. Third, due to the lower spatial resolution and lower signal noise ratio in IVIM-DWI and its parameter mappings, only ROIs based analysis had been performed. At last, only the intramuscular tumors were evaluated in this study. Further research is needed to determine the depth and location of tumors (subcutaneous, intramuscular or intraperitoneal), size of tumors, and size of the ablative lesions that may have any influence on IVIM-DWI monitoring.

## Conclusion

The therapeutic effect induced by RF Ablation treatment can be effectively evaluated with IVIM-derived parameters. The IVIM-DW derived perfusion parameters have the potential to indicate therapy effects immediately after RF Ablation treatment without significant association with classical tumor perfusion metrics derived from DCE-MRI and perfusion CT measurements.
